# Loss of PTEN is associated with elevated EGFR and HER2 expression and worse prognosis in salivary gland cancer

**DOI:** 10.1038/bjc.2011.605

**Published:** 2012-01-12

**Authors:** T Ettl, K Baader, C Stiegler, M Müller, A Agaimy, J Zenk, T Kühnel, M Gosau, K Zeitler, S Schwarz, G Brockhoff

**Affiliations:** 1 Department of Oral and Maxillofacial Surgery, University of Regensburg, Franz-Josef-Strauss-Allee 11, 93053 Regensburg, Germany; 2 Department of Pathology, University of Regensburg, Franz-Josef-Strauss-Allee 11, 93053 Regensburg, Germany; 3 Department of Pathology, University of Erlangen-Nuremberg, Krankenhausstraße 12, 91054 Erlangen, Germany; 4 Department of Otorhinolaryngology, University of Erlangen-Nuremberg, Waldstraße 1, 91054 Erlangen, Germany; 5 Department of Otorhinolaryngology, University of Regensburg, Franz-Josef-Strauss-Allee 11, 93053 Regensburg, Germany; 6 Department of Gynecology and Obstetrics, University of Regensburg, Landshuter Straße 65, 93053 Regensburg, Germany

**Keywords:** salivary gland cancer, PTEN, deletion, prognosis, EGFR HER2

## Abstract

**Background:**

Activity of the tumour-suppressor gene PTEN is reduced in different types of cancer and implicates non-responsiveness to targeted therapy. This study evaluates the gene and protein status of PTEN in salivary gland carcinomas.

**Methods:**

A total of 287 carcinomas of the major and minor salivary glands were investigated for phosphatase and tensin homologue located on chromosome 10 (PTEN) deletion and loss of PTEN expression using fluorescence *in situ* hybridisation (FISH) and immunohistochemistry (IHC), respectively. Results were correlated to clinicopathological parameters, long-term survival, epidermal growth factor receptor (EGFR) and human epidermal growth factor receptor 2 (HER2) (IHC and FISH) status of the tumours.

**Results:**

Hemizygous deletions of PTEN were found in 35 out of 232 (15.1%) carcinomas, while homozygous deletions were observed in 17 out of 232 (7.3%) tumours. Phosphatase and tensin homologue located on chromosome 10 deletion was common in certain histological subtypes and especially homozygous deletion was associated with high-grade malignancy, lymph node metastases and unfavourable long-term prognosis (*P*<0.001). Loss of PTEN expression was present in 59 out of 273 (21.6%) carcinomas and was significantly correlated to genomic PTEN deletion, high-grade malignancy (*P*<0.001), increased tumour size (*P*=0.036), lymph node metastases (*P*=0.007) and worse disease-specific survival (*P*=0.002). Genomic PTEN deletion, in particular homogenous deletion (*P*<0.001) predominantly occurred in tumours with increased gene copy number of EGFR (60.0%) and/or amplification of HER2 (63.6%). Loss of PTEN expression was frequently found in tumours overexpressing EGFR (28.6%) and/or HER2 (52.6%).

**Conclusion:**

PTEN function is reduced in different types of salivary gland cancer indicating unfavourable prognosis. Its association with EGFR and HER2 signalling might affect targeted therapy.

Salivary gland carcinomas are rare tumours with an annual incidence rate between 0.4 and 2.6 per 100 (Eveson *et al*, 2005). As the current World Health Organisation (WHO) classifies 24 different malignant subtypes with varying clinical courses and prognoses, diagnosis and treatment of these tumours represent a special challenge for both pathologists and surgeons. Recent attempts of treatment for unresectable or metastatic disease comprise targeted therapies against the epidermal growth factor receptor (EGFR, ErbB1) and the human epidermal growth factor receptor 2 (HER2, ErbB2) (Haddad *et al*, 2003; Agulnik *et al*, 2007; Locati *et al*, 2008). To date, however, response rates are rather disappointing.

Phosphatase and tensin homologue located on chromosome 10 (PTEN) is a tumour-suppressor gene frequently lost on chromosome 10q23 in different types of cancer (Li *et al*, 1997). In the nucleus, PTEN regulates genomic stability, cell cycle progression, differentiation and gene expression (Carracedo *et al*, 2011). Beside these tumour-suppressive activities, PTEN functions as a negative regulator of the PI3K pathway by dephosphorylating the 3′ position of phosphoinositide 3,4,5-triphosphate (PIP3). Stimulated by EGFR or HER2, PI3K activates the Ser/Thr kinase AKT, thus promoting cell survival and tumour growth (Courtney *et al*, 2010). Hemi- or homozygous deletions of PTEN are frequent events in prostate cancer and high-grade gliomas with unfavourable impact on prognosis (Korshunov *et al*, 2004; Yoshimoto *et al*, 2007). Loss of PTEN seems associated with unresponsiveness to targeted therapy against EGFR in colorectal cancer and glioblastomas (Mellinghoff *et al*, 2005; Frattini *et al*, 2007; Negri *et al*, 2010), moreover it predicts resistance to trastuzumab in HER2-positive breast cancer (Nagata *et al*, 2004; Pandolfi, 2004). The purpose of this study was to evaluate the gene and protein status of PTEN in a representative cohort of salivary gland carcinomas and to correlate the results to clinicopathological parameters, to long-term survival, and to the gene and protein status of EGFR and HER2.

## Patients and methods

### Patients and treatment modalities

The study comprised 287 patients with carcinomas of the major and minor salivary glands, diagnosed at the Departments of Pathology of the University Hospital Regensburg, the University Hospital Erlangen-Nuremberg and the Hospital Clinic Nuremberg between 1984 and 2008. Clinical and follow-up data were obtained from the clinical tumour registries of Regensburg and Erlangen-Nuremberg in accordance with the Research Ethic Guidelines of the medical faculties. At diagnosis, patients were staged according to the TNM system of the Union for International Cancer Control (UICC) (Sobin *et al*, 2009). Tumour surgery was performed at the Departments of Oral and Maxillofacial Surgery and Otorhinolaryngology of Regensburg University Hospital, Erlangen-Nuremberg University Hospital and Nuremberg City Hospital. A total of 287 patients underwent primary surgery; lymph node dissection was performed for 205 (71.9%) patients. Postoperative radio- or radiochemotherapy was applied in 170 (59.2%) cases with high-grade malignancy, positive resection margins, lymph node metastases or distant metastasis.

A total of 204 (71.1%) parotid, 39 (13.6%) submandibular, 2 (0.7%) sublingual and 42 (14.6%) minor gland carcinomas were recorded. In all, 56.1% of the patients presented with advanced (III, IV) UICC tumour stages at diagnosis. Cervical lymph node metastases were obvious in 31.8%, distant metastases occurred in 10.6% of the patients. In 217 cases (77.8%), close resection margins (R0) were achieved, 48 (17.2%) patients ended up with microscopic (R1) and 14 (5.0%) patients with macroscopic (R2) residual tumour after surgery. With view to the tumours originating from the minor salivary glands clear margins were achieved in 73.8% (31 out of 42). Ten (23.8%) carcinomas presented microscopic residual tumour and one minor gland carcinoma (2.4%) ended up with macroscopic residual tumour after surgery.

### Follow-up studies

In total, 137 male and 150 female patients showed a mean age of 60.6 (11–99) years at diagnosis. The mean follow-up of all patients was 4.75 (range 0.1–21.2) years. Recurrence was observed in 74 (26.8%) patients. Disease-related deaths occurred in 73 (25.4%) cases. The 5- and 10-year disease-specific survival rates of all patients were 73.2% and 68.1%, respectively.

### Histology and classification

Haematoxylin-eosin slides from paraffin wax-embedded tumours were available for all cases and were independently reviewed by two experienced pathologists (SS and AA) without knowledge of the initial diagnosis. All tumours were classified according the contemporary WHO's classification of salivary gland tumours (Barnes *et al*, 2005). The study cohort comprised 40 acinic cell carcinomas (ACCCs), 52 adenoid cystic carcinomas (ACCs), 45 mucoepidermoid carcinomas (MECs), 27 salivary duct carcinomas (SDCs), 31 adenocarcinomas NOS (ACNOS), 28 squamous cell carcinomas (SQCCs), 21 myoepithelial carcinomas (MYECs), 10 polymorphous low-grade adenocarcinomas (PLGAs), 9 basal cell adenocarcinomas (BCACs), 9 oncocytic carcinomas (OCCs), 4 epithelial–myoepithelial carcinomas (EMCs), 4 malignant mixed tumours (MMTs), 4 undifferentiated carcinomas (UCs), 2 large cell carcinomas (LCCs) and 1 cystadenocarcinoma (CACs). The less frequent entities PLGA, BCAC, OCC, EMC, MMT, UC, LCC and CAC were summarised as ‘others’ in Table 1. All cases of SQCC were classified as primitive of the salivary glands after intensive staging procedures (CT or MRI of the head and neck, panendoscopy, X-ray or CT of the chest and ultrasonograpy of the abdomen) and exclusion of a metastasis to the salivary gland. Squamoid variants of MECs were thoroughly sorted out (Schwarz *et al*, 2011). Grading was based on a three-tiered grading system (Therkildsen *et al*, 1998; Jouzdani *et al*, 2010). The ACCC, BCAC, EMC, CAC and PLGA were considered low grade (G1) with the exception of dedifferentiated tumours, which were classified high grade (G3). In contrast, SDC, SQCC, MMT, OCC, UC and LCC were classified high grade (G3). Mucoepidermoid carcinomas were graded according to the criteria proposed in the current WHO classification (Barnes *et al*, 2005). Adenoid cystic carcinomas were divided into predominantly tubulo-cribriform (G2) and predominantly solid (G3) tumours. Grading of ACNOS and MYEC was based on nuclear pleomorphism and mitotic activity similar to the Elston and Ellis grading of breast cancer (Elston and Ellis, 1991). The 27 cases of carcinoma ex pleomorphic adenoma were classified and graded according to the malignant component of the tumour.

### Immunohistochemistry

A tissue microarray (TMA) with 2.0 mm diameter punch cores was constructed from formalin-fixed paraffin-embedded tissue blocks of all patients as previously described (Milanes-Yearsley *et al*, 2002). Haematoxylin–eosin-stained TMA sections were used for reference histology.

**Figure 1:**
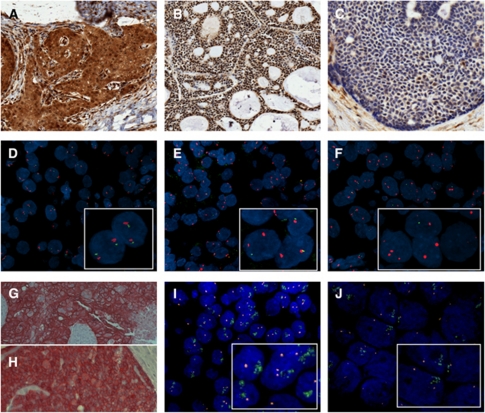
(**A**) Strong nuclear and cytoplasmic PTEN immunostaining in ACCC (IRS 300, × 200). (**B**) Strong nuclear PTEN staining in ACC (IRS 300, × 100), (**C**) Weak nuclear PTEN staining in ACC (IRS 10, × 200). (**D**) Disomy of genomic PTEN in ACCC. (**E**) Hemizygous deletion of PTEN in ACCC. (**F**) Homozygous deletion of PTEN in SDC. (**G**) 3+ staining of HER2 in SDC ( × 200). (**H**) 3+ staining of EGFR in ACC ( × 200). (**I**) Cluster amplification of HER2 in SDC. (**J**) Cluster amplification of EGFR in MEC.

Immunostaining of PTEN (Cell Signaling Technology, Inc., Danvers, MA, USA, 138G6, #9559, monoclonal rabbit, dilution 1 : 50, detection EnVision Dual Link System, Dako, Glostrup, Denmark), EGFR (Dako, EGFR pharmDx, clone 2-18C9, monoclonal mouse, dilution 1 : 400, detection En Vision, Dako) and HER2 (Dako, A0485, polyclonal rabbit, dilution 1 : 250, detection iVIEW DAB, Ventana Medical Systems, Inc., Illkirch, France) was performed on 5 *μ*m sections of the TMAs and applied according to the manufacturer's instructions. After dewaxing, washing and rehydration of the slides through xylene and graded alcohols, microwave heating in citrate buffer was used for antigen retrieval. In case of EGFR, proteinase K was applied for epitope retrieval. Endogenous peroxidase was blocked in ChemMate peroxidase-blocking solution (Dako). Immunohistochemistry (IHC) for PTEN was semiquantitatively evaluated based on nuclear and cytoplasmic staining (Figure 1A–C). An immunoreactive score (IRS) was built as the product of staining intensity (none=0, weak=1, moderate=2, strong=3) and the percentage of positive tumour cells (0–100%) resulting in an IRS ranging from 0 to 300 points (Laurent-Puig *et al*, 2009). Tumours were dichotomised into PTEN-negative (IRS 0–59) and PTEN-positive (IRS 60–300). The immunostaining of EGFR and HER2 was semiquantitatively evaluated based on intensity of membrane reactivity following the original DAKO Herceptest criteria with a threshold of 10% immunopositive cells: 0, negative (no reactivity or reactivity in <10 of cells); 1+, weak reactivity in >10% of cells, 2+ moderate reactivity in >10% cells; 3+ strong reactivity in >10% cells. Tumours with 3+ staining were considered as positive (Figure 1G and H). Immunostaining pattern was also documented in normal salivary gland tissues as controls.

### Fluorescence *in situ* hybridisation

As described in detail elsewhere (Sassen *et al*, 2008) TMA sections were mounted on charged slides (SuperFrost Plus; Menzel GmbH, Braunschweig, Germany). Haematoxylin–eosin-stained TMA sections were used for reference histology. Fluorescence *in situ* hybridisation (FISH) was performed with the use of directly labelled Zyto*Light* SPEC PTEN/CEN10, SPEC EGFR/CEN7 and SPEC HER2/CEN17 dual colour probes (ZytoVision Ltd, Bremerhaven, Germany). After probe, hybridisation nuclei were counterstained with anti-fading 4′,6-diamidino-2-phenylindole Vectashield (Vector Laboratories, Burlingame, CA, USA) and were analysed by epifluorescence microscopy using the AxioImager-Z1 (Zeiss, Göttingen, Germany). Hybridisation signals of 50 non-overlapped nuclei were manually counted on single cell basis by two independent observers. Non-neoplastic salivary gland specimens were used as controls.

Homozygous deletion of PTEN was defined by the simultaneous lack of both PTEN locus signals and by the presence of centromere signals in >20% of nuclei. Hemizygous deletion of PTEN was defined as >30% of tumour nuclei containing either one PTEN locus signal and ⩾2 centromere signals or 2 PTEN locus signals and ⩾4 centromere signals (relative deletions) (Sircar *et al*, 2009). Representative examples are shown in Figures 1D–F (centromere signals in red).

For EGFR, samples were grouped as normal disomy, ⩽2 centromere signals in ⩾50% of cells; low polysomy/trisomy, ⩾3 centromere signals in ⩾40% of cells, excluding cases with high polysomy or gene amplification; high polysomy, ⩾4 centromere signals in ⩾40% of cells, excluding cases with gene amplification; and gene amplification, ratio of gene/chromosome ⩾2 or clusters of probes (>10 copies per tumour cell) in ⩾40% of cells. Disomy and trisomy/low polysomy were grouped as FISH negative, while high polysomy and amplification (Figure 1J) were classified as FISH positive or copy number gain (CNG) of EGFR in dichotomisation (Chung *et al*, 2006; Pectasides *et al*, 2011).

Human epidermal growth factor receptor 2 was evaluated referring to the guideline recommendations for HER2 testing in breast cancer (Wolff *et al*, 2007). Gene amplification was assessed by a FISH ratio (HER2 gene signals to chromosome 17 signals) of >2.2 (Figure 1I).

### Statistical analysis

Data were analysed with SPSS for Windows, version 15.0 (SPSS, Erkrath, Germany). Relationships between parameters were examined using Pearson's *χ*^2^-test (*P*<0.05) and Fisher's exact probability test (*P*<0.05) for dichotomised variables. Univariate survival curves were calculated by the Kaplan–Meier method and distributions were compared using the log-rank test. Disease-specific overall survival was calculated from the date of diagnosis until disease-caused death or end of follow-up. Cox proportional hazards model (enter method) was used in multivariate analyses.

## Results

### PTEN FISH analysis

A total of 232 salivary gland carcinomas were available for PTEN FISH analysis (Table 1). Hemizygous deletion was found in 35 (15.1%) tumour samples and homozygous deletion was identified in 17 (7.3%) carcinomas resulting in an overall deletion rate of 22.4% (*n*=52). Representative images are shown in Figure 1. Hemi- and homozygous deletions of PTEN were commonly found in SDCs (66.6%), ACNOS (50%) and SQCCs (42.3%). One out of three SDCs presented a homozygous deletion. In contrast, deletions were rare events in ACC and MEC (Table 1). Genomic loss of PTEN (hemi- and homozygous deletion) was associated with high-grade malignancy (*P*<0.001), lymph node metastases (*P*<0.001) and advanced (III, IV) tumour stage (*P*=0.002). Homozygous PTEN deletion displayed more aggressive behaviour as 94.1% (16 out of 17) of these tumours were high-grade carcinomas in contrast to 60.0% (21 out of 35) high-grade malignancies among the tumours with hemizygous deletion of PTEN.

In all, 60.0% (18 out of 30) of the tumours with an increased EGFR copy number presented hemi- or homozygous deletion of PTEN (87.5% of the SDCs) in contrast to only 17.1% (27 out of 158) PTEN deletion in EGFR-negative tumours (*P*<0.001). Phosphatase and tensin homologue located on chromosome 10 deletion was also associated with EGFR protein overexpression (15 out of 42, 35.7% *vs* 30 out of 153, 19.6% of EGFR-negative tumours, *P*=0.038). In total, 63.6% (7 out of 11) of tumours with an amplification of HER2 presented a deletion (6 homozygous) of PTEN (83.3% of the SDCs) in comparison with only 20% (37 out of 185) PTEN deletions in the non-amplified carcinomas (*P*<0.001). Moreover, PTEN deletion was very commonly found (*P*<0.001) in tumours with HER2 overexpression (13 out of 18, 72.2%), while rarely detected in HER2-negative tumours (32 out of 177, 18.1%). Considering all tumours indicative for anti-HER2 therapy (overexpression and amplification), 14 out of 23 (60.8%) showed a genomic loss of PTEN (*P*<0.001). Homozygous deletion of PTEN was stronger associated with EGFR CNG or HER2 amplification (10 out of 16, 62.5% and 6 out of 16, 37.5%, respectively) than hemizygous deletion (8 out of 29, 27.6% and 1 out of 28, 3.6%, respectively, Table 1).

**Figure 2:**
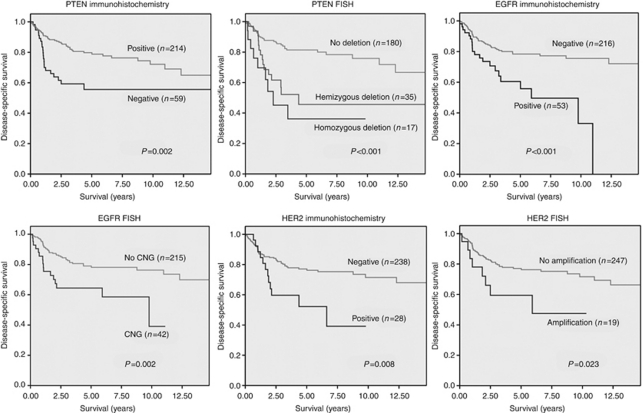
Univariate Kaplan–Meier survival analyses for IHC and FISH of PTEN, EGFR and HER2.

Deletion of genomic PTEN was strongly associated with immunohistochemical loss of PTEN expression (*P*<0.001). In all, 50.0% (24 out of 48) of the tumours with PTEN deletion (48.5% of hemizygous and 53.3% of homozygous deletion) also indicated loss of PTEN protein expression in contrast to 14.1% (24 out of 170) PTEN expression loss in non-deleted cases. Hemi- and homozygous deletion of PTEN were associated with lower disease-specific survival rates in univariate Kaplan–Meier analysis revealing even worse prognosis for homozygous deletion (Figure 2). Prognostic significance of genomic PTEN was independent of grading, EGFR and HER2 status. Deletion of PTEN (hemi- and homozygous) harboured an unfavourable outcome in both EGFR-negative (*P*<0.001) and -positive cases (*P*=0.039). Similarly, loss of PTEN indicated worse prognosis in HER2-negative (*P*=0.003) and HER2-positive (amplification and overexpression, *P*=0.027) tumours. With regard to grading, PTEN deletion went along with lower survival rates in both low/intermediate grade (*P*=0.002) and high-grade tumours (*P*=0.120), although not reaching statistical significance in the latter category. Moreover, deletion of PTEN (hemi- and homozygous) evolved as a strong negative predictor from multivariate Cox regression analysis (Table 2).

### PTEN IHC

Results of combined nuclear and cytoplasmic PTEN staining (*n*=273) are shown in Table 1. The average PTEN IRS was 122 (range 0–300). Negative PTEN expression (IRS<60) was identified in 21.6% (*n*=59) of all tumours and was most frequently found in SDCs (41.7%), SQCCs (37.0%) and ACNOS (29.6%). Loss of PTEN expression was associated with advanced age (*P*=0.005), high-grade malignancy (*P*<0.001), increased tumour size (*P*=0.036), lymph node metastases (*P*=0.007) and advanced (III, IV) tumour stage (*P*=0.004).

Loss of PTEN expression was also correlated to an increased gene copy number of EGFR, because 45.5% (15 out of 33) of the tumours with a CNG of EGFR presented a negative PTEN expression in comparison with 16.4% (30 out of 83) PTEN negativity in tumours without a CNG of EGFR (*P*=0.001). Negative PTEN expression more frequently occurred in carcinomas overexpressing EGFR protein (14 out of 49, 28.6%) than in those without EGFR overexpression (35 out of 188, 18.6%), although not reaching statistical significance (*P*=0.164). In all, 10 out of 19 (52.6%) carcinomas with an overexpression of HER2 protein showed an immunohistochemical loss of PTEN in comparison with 17.5% (38 out of 217) PTEN negativity in HER2-negative tumours (*P*<0.001). Phosphatase and tensin homologue located on chromosome 10 was more often absent in HER2-amplified tumours (4 out of 11, 36.4%) than in those without HER2 amplification (43 out of 214, 20.1%, *P*=0.247). In univariate analysis, loss of PTEN expression was significantly associated with unfavourable long-term survival (*P*=0.002, Figure 2).

### EGFR and HER2

Fluorescence *in situ* hybridisation analysis for EGFR was available for 257 cases. In all, 42 (16.3%) tumour specimens presented an increased gene copy number (39 high polysomies and 3 amplifications). Epidermal growth factor receptor positivity was associated with high-grade carcinomas (*P*<0.001), tumour-size (*P*=0.003), lymph node metastases (*P*=0.003) and advanced (III, IV) tumour stage (*P*<0.001). In univariate Kaplan–Meier analysis CNG of EGFR predicted worse survival (*P*=0.002, Figure 2). Immunohistochemical analysis of EGFR was available for 269 cases. In total, 53 tumours (19.7%) presented overexpression of EGFR. Epidermal growth factor receptor positivity was associated with age >70 (*P*<0.001), high-grade malignancy (*P*=0.001) and worse overall survival in univariate analysis (*P*<0.001, Figure 2). Increased EGFR copy number was strongly correlated to EGFR overexpression (*P*<0.001).

Human epidermal growth factor receptor 2 FISH analysis was performed for 266 cases. In 19 cases (7.1%), amplification (11 SDCs) occurred and was associated with positive lymph nodes (*P*=0.008), high-grade malignancy (*P*<0.001) and unfavourable overall survival (*P*=0.023, Figure 2).

In all, 28 out of 266 (10.5%) investigated cases (16 SDCs) presented immunohistochemical overexpression of HER2. Human epidermal growth factor receptor 2 positivity was associated with positive lymph nodes (*P*<0.001), advanced tumour stage (*P*=0.004), high-grade malignancy (*P*<0.001) and worse survival (*P*=0.008, Figure 2). Human epidermal growth factor receptor 2 amplification was strongly associated with HER2 overexpression (*P*<0.001). Moreover, HER2 amplification was associated with CNG of EGFR (*P*=0.013).

## Discussion

This study investigated the role of the tumour-suppressor PTEN in a considerable and representative number of salivary gland cancer. By use of interphase FISH analysis, it revealed for the first time a hemi- or homozygous deletion of the gene on chromosome 10q23.3 in >20% of these tumours, especially in SDCs, ACNOS and SQCCs. Homozygous deletion of PTEN (7.3% of all tumours) was almost exclusively (94.1%) found in high-grade malignancies pointing towards a more aggressive growth pattern in comparison with hemizygous gene deletion. This observation is in accordance with the hypothesis that hemizygous loss of PTEN with haplo-insufficiency of the remaining allele leads to genomic instability and cancer development as shown by *in vivo* studies on knockout PTEN mice (Di Cristofano *et al*, 1998; Kwabi-Addo *et al*, 2001; Shen-Li *et al*, 2011), while complete inactivation accelerates tumour dedifferentiation, progression and metastases (Verhagen *et al*, 2006; Schmitz *et al*, 2007). In our study, both hemi- and homozygous genomic deletion of PTEN strongly correlated with immunohistochemical loss of the protein expression. Loss of PTEN function, especially deletion of genomic PTEN independently indicated an unfavourable clinical course of patients with lymph node metastases, rapid tumour progression and worse overall survival. In line with the above mentioned hypothesis, homozygous deletion displayed an even worse prognosis compared with hemizygous deletion in the investigated salivary gland carcinomas. Downregulation of PTEN with negative impact on prognosis has already been described for prostate cancer, colorectal and breast cancer and high-grade gliomas (Korshunov *et al*, 2004; Fujita *et al*, 2006; Frattini *et al*, 2007; Yoshimoto *et al*, 2007; Laurent-Puig *et al*, 2009).

Different types of salivary gland carcinomas show overexpression and an increased gene copy number of EGFR and HER2 with negative impact on prognosis (Press *et al*, 1994; Ettl *et al*, 2008; Lujan *et al*, 2010; Williams *et al*, 2010). In this study, 16.3% of the carcinomas presented high polysomy or amplification of the EGFR gene and 19.7% showed overexpression of the EGFR protein. Overexpression and amplification of HER2 occurred in 10.5% and 7.1% of cases, respectively, especially in SDCs. Both EGFR and HER2 were associated with negative clinicopathological parameters and unfavourable survival. Increased gene copy number of EGFR and overexpression/amplification of HER2 pose the precondition for anti-EGFR and anti-Her2 therapy in breast, colorectal and lung cancer (Wolff *et al*, 2007; Hirsch *et al*, 2008; Laurent-Puig *et al*, 2009). Moreover, in salivary gland cancer first phase-II trials on targeted therapies have been conducted and revealed that the use of anti-EGFR agents, such as cetuximab and gefitinib, failed to produce obvious response, although the majority of patients showed disease stabilisation (Glisson *et al*, 2005; Locati *et al*, 2009). These studies mainly comprised adenoid cystic carcinomas (68–77%) and the EGFR status was characterised by IHC. In the investigation of Locati *et al* (2009), tumours were also retrospectively analysed by FISH, however, no amplification or high polysomy of EGFR was found in these tumours. This might have been a reason for missing response, as EGFR protein expression observed by IHC has been described as an unreliable predictor of responsiveness to EGFR inhibitors (Ciardiello and Tortora, 2008; Vidal *et al*, 2009). The use of trastuzumab (HER2-inhibitor) and lapatinib (combined TKI against EGFR and HER2) did also show no obvious reactivity in ACCs (Haddad *et al*, 2003; Agulnik *et al*, 2007; Vidal *et al*, 2009). However, partial response to trastuzumab was reported for a mucoepidermoid carcinoma with HER2 3+ immunostaining (Haddad *et al*, 2003) and prolonged tumour progression to lapatinib was documented in three non-ACC tumours with HER2 amplification and 3+ staining for EGFR and HER2 (Vidal *et al*, 2009). Therefore, increased gene copy number of EGFR and amplification or overexpression of HER2 should be a precondition for an anti-EGFR and anti-HER2 therapy in salivary gland cancer, too.

In this study, we demonstrate that deletion of genomic PTEN, in particular homogenous deletion, predominantly occurs in salivary gland carcinomas with an increased copy number of EGFR or amplification of HER2. Moreover, loss of PTEN protein expression is frequently found in tumours with EGFR and HER2 overexpression. This coincidence of PTEN loss and EGFR/HER2 gene/expression gain most likely represents an accumulation of independent genetic disorders in poorly differentiated carcinomas during tumourigenesis rather than a functional relationship between EGFR, HER2 and PTEN. Owing to its function as a tumour suppressor, however, there is some evidence that PTEN has significant impact on the efficiency of targeted therapies by antagonising the activation of the PI3K–AKT pathway that is predominantly activated by EGF-/HER2-receptor tyrosine kinases in different tumour types. In successfully treated cancers, PI3K signalling is attenuated or even turned off by EGFR- or HER2-targeting (using antibodies or receptor kinase inhibitors). If there is a loss of PTEN expression/function, however, PI3K signalling remains active in spite of anti-EGFR or anti-HER2 therapy (Courtney *et al*, 2010; Nahta and O'Regan, 2010). In breast cancer, (active), PTEN has been reported to be an essential parameter for responsiveness to trastuzumab treatment, while loss of PTEN rather predicts resistance to trastuzumab in HER2-positive patients (Nagata *et al*, 2004; Pandolfi, 2004; Fujita *et al*, 2006). On the other hand, the additional use of a PI3K inhibitor is reported to overcome the PTEN loss-induced trastuzumab resistance (Courtney *et al*, 2010). In colorectal cancer, high polysomy or amplification of EGFR was associated with response to cetuximab therapy, whereas loss of PTEN expression, (evaluated by IHC) rather is indicative for therapy resistance (Frattini *et al*, 2007; Laurent-Puig *et al*, 2009; Loupakis *et al*, 2009). The same observation was made in glioblastomas, where expression of PTEN was associated with clinical response to the EGFR kinase inhibitors gefitinib and erlotinib (Mellinghoff *et al*, 2005). For these tumours, evidence derived from *in vitro* analysis suggests that resistance can be overcome by coupling anti-EGFR-agents with mTOR inhibitors (Wang *et al*, 2006).

In anti-EGFR/Her2-treated salivary gland cancer patients, a retrospective analysis could elucidate the suggested correlation of the PTEN status with therapy response rates. As a consequence, PTEN analysis might find the way into routine diagnostics and might facilitate therapy decisions related to anti-EGFR or anti-HER2 targeting. Additional targeting of the PI3K pathway potentially enhances therapy efficiency in salivary gland cancer.

Despite the significant findings in this study it has to be kept in mind that the investigated salivary gland carcinomas we investigated comprise a variety of subtypes with inter- and intra-tumoural differences and characteristics. This fact aggravates further preclinical and clinical investigations of these rare tumours.

In conclusion, this is the first report on a reduced PTEN function in different types of salivary gland cancer indicating worse prognosis. We could demonstrate an association between PTEN loss and EGFR and HER2 signalling, which might influence response to targeted therapies.

## Figures and Tables

**Table 1:** PTEN and clinicopathological parameters

	**PTEN FISH**			**PTEN IHC**	
**Parameter**	**Non-deleted**	**Hemizygous**	**Homozygous**	**Pos**	**Neg**
Total	180 (77.5%)	35 (15.1%)	17 (7.3%)	214 (78.4%)	59 (21.6%)
ACCC	30 (85.7%)	5 (14.3%)	0	36 (90.0%)	4 (10.0%)
ADCC	41 (97.6%)	1 (2.4%)	0	42 (84.0%)	8 (16.0%)
MEC	35 (89.7%)	3 (7.7%)	1 (2.6%)	36 (85.7%)	6 (14.3%)
SDC	8 (33.3%)	9 (37.5%)	7 (29.2%)	14 (58.3%)	10 (41.7%)
ACNOS	11 (50.0%)	5 (22.7%)	6 (27.3%)	19 (70.4%)	8 (29.6%)
SQCC	15 (57.7%)	9 (34.6%)	2 (7.7%)	17 (63.0%)	10 (37.0%)
MYEC	15 (83.3%)	2 (11.1%)	1 (5.6%)	19 (95.0%)	1 (5.0%)
Others	25 (13.9%)	1 (2.7%)	0	31 (72.1%)	12 (27.9%)
					
*Age*					
<70	108 (82.4%)	15 (11.5%)	8 (6.1%)	**140** (**84.3%)**	**26** (**15.7%)**
>70	48 (68.6%)	14 (20.0%)	8 (11.4%)	**74** (**69.2%)**	**33** (30.8%)**
					
*Grade*					
Low/intermediate	**112** (**88.2%)**	**14** (**11.0%)**	**1** (**0.8%)**	**131** (**86.8%)**	**20** (**13.2%)**
High	**68** (**64.8%)**	**21** (**20.0%)**	**16** (15.2%)***	**83** (**68.0%)**	**39** (32.0%)***
					
*T-stage*					
T1–T2	103 (79.2%)	19 (14.6%)	8 (6.2%)	**130** (**82.8%)**	**27** (**17.2%)**
T3–T4	73 (74.5%)	16 (16.3%)	9 (9.2%)	**80** (**71.4%)**	**32** (28.6%)*
					
*N-stage*					
N0	**134** (**85.4%)**	**16** (**10.2%)**	**7** (**4.5%)**	**152** (**82.6%)**	**32** (**17.4%)**
N1–3	**39** (**57.4%)**	**19** (**27.9%)**	**10** (14.7%)***	**55** (**67.1%)**	**27** (32.9%)**
					
*EGFR FISH*					
No CNG	**131** (**82.9%)**	**21** (**13.3%)**	**6** (**3.8%)**	**153** (**83.6%)**	**30** (**16.4%)**
CNG	**12** (**40.0%)**	**8** (**26.7%)**	**10** (33.3%)***	**18** (**54.5%)**	**15** (45.5%)***
					
*EGFR IHC*					
0+,1+,2+	**123** (**80.4%)**	**20** (**13.1%)**	**10** (**6.5%)**	153 (81.4%)	35 (18.6%)
3+	**27** (**64.3%)**	**9** (**21.4%)**	**6** (14.3%)*	35 (71.4%)	14 (28.6%)
					
*HER2 FISH*					
No amplification	**148** (**80.0%)**	**27** (**14.6%)**	**10** (**5.4%)**	171 (79.9%)	43 (20.1%)
Amplification	**4** (**36.4%)**	**1** (**9.1%)**	**6** (54.5%)***	7 (63.6%)	4 (36.4%)
					
*HER2 IHC*					
0, 1+, 2+	**145** (**81.9%)**	**23** (**13.0%)**	**9** (**5.1%)**	**179** (**82.5%)**	**38** (**17.5%)**
3+	**5** (**27.8%)**	**6** (**33.3%)**	**7** (38.9%)***	**9** (**47.4%)**	**10** (52.6%)***

Abbreviations: ACC=adenoid cystic carcinoma; ACNOS=adenocarcinoma NOS; CNG=copy number gain; EGFR=epidermal growth factor receptor; FISH=fluorescence *in situ* hybridisation; HER2=human epidermal growth factor receptor 2; IHC=immunohistochemistry; MEC=mucoepidermoid carcinoma; MYEC=myoepithelial carcinoma; Neg=negative; Pos=positive; PTEN=phosphatase and tensin homologue located on chromosome 10; SDC=salivary duct carcinoma; SQCC=squamous cell carcinoma.

^*^P⩽0.05, ^**^P⩽0.01, ^***^P⩽0.001. Statistically significant associations are highlighted in bold.

**Table 2:** Univariate (Kaplan-Meier – log-rank) and multivariate (Cox regression – Enter) analysis

		**Univariate**	**Multivariate**	
**Variable**	**Coding**	**Log-rank**	**Significance**	**HR (95% CI)**
Grade	G1/G2 *vs* G3	**<0.001**	0.052	2.56 (0.99–6.60)
T-stage	1, 2 *vs* 3, 4	**<0.001**	**0.029**	2.50 (1.10–5.69)
N-stage	0 *vs* 1, 2, 3	**<0.001**	0.061	2.06 (0.97–4.38)
R-stage	R0 *vs* R1/R2	**<0.001**	**0.004**	3.03 (1.43–6.43)
EGFR FISH	No CNG *vs* CNG	**0.002**	0.964	1.02 (0.40–2.64)
EGFR IHC	Neg *vs* Pos	**<0.001**	0.111	1.94 (0.86–4.39)
HER2 FISH	No Ampl *vs* Ampl.	**0.023**	0.634	0.71 (0.18–2.89)
HER2 IHC	Neg *vs* Pos	**0.008**	0.301	0.60 (0.23–1.58)
PTEN FISH	No Del *vs* Del	**<0.001**	**0.012**	3.17 (1.29–7.78)
PTEN IHC	Pos *vs* Neg	**0.002**	0.397	1.49 (0.59–3.77)

Abbreviations: Ampl=amplification; CI=confidence interval; CNG=copy number gain; Del=deletion; EGFR=epidermal growth factor receptor; FISH=fluorescence *in situ* hybridisation; HER2=human epidermal growth factor receptor 2; HR=hazard ratio; IHC=immunohistochemistry; n=number of patients; Neg=negative; Pos=positive; PTEN=phosphatase and tensin homologue located on chromosome 10; R=residual tumour. Statistically significant associations are highlighted in bold.
